# Studies on the Synthesis Process of Plant-Derived Ursodeoxycholic Acid Intermediates

**DOI:** 10.3390/molecules30071454

**Published:** 2025-03-25

**Authors:** Shaoxiong Jing, Zhongyue Wang, Yuan Wang, Yingquan Yang, Jian Song, Bao Zhang

**Affiliations:** 1 School of Chemical Engineering and Technology, Tianjin University, Tianjin 300350, China; jsx_2022@tju.edu.cn (S.J.); yue7946801@tju.edu.cn (Z.W.); wangyuan@tju.edu.cn (Y.W.); 2 Suzhou Entai New Materials Technology Company, Suzhou 215124, China; okyangyq@outlook.com

**Keywords:** ursodeoxycholic acid, synthesis process optimization, impurity studies

## Abstract

Ursodeoxycholic acid (UDCA), a critical secondary bile acid in human physiology, demonstrates significant industrial potential through synthetic routes from bisnoralcohol (BA). Current synthetic routes rely on hydroxyl oxidation and Horner–Wadsworth–Emmons reactions as critical initial steps, facing unresolved challenges in reaction scale-up dynamics and impurity evolution. In this work, we systematically investigated the scale-up effects and innovatively addressed the impurity control problem. In the OH-C(22) selective oxidation of BA, the impurity C(22) carboxylic acid was synthesized, the emulsification was eliminated by process optimization, and the yield was increased from 89.0% to 95.2%. In the Horner–Wadsworth–Emmons reaction, the C(20)-methyl racemate and the C(22)-Z-ene isomer were synthesized, followed by the validation of the remaining byproducts. Based on impurity profile analysis, we innovatively modified the reaction feeding protocol, increased the yield from 79.1% to 90.8%, and significantly improved reaction selectivity. This optimized process demonstrates superior scalability and provides valuable insights for the industrial production of plant-derived UDCA.

## 1. Introduction

As a significant secondary bile acid present in human bile [1], Ursodeoxycholic acid (UDCA) can reduce cholesterol and cholesteryl ester content in bile, promoting the gradual dissolution of cholesterol in gallstones. It is the only bile-acid-based drug approved by the Food and Drug Administration (FDA) for the non-surgical treatment of cholesterol gallstones [2,3,4]. UDCA also exhibits various biological activities, including non-surgical treatment of cholesterol gallstones, anti-apoptotic effects, anti-inflammatory properties, and antitumor activity [5,6,7,8,9,10]. In the early stages, UDCA was primarily extracted from bile drained from live bear gallbladders. However, this method cannot meet industrial production demands and violates animal protection laws. Currently, UDCA production methods mainly rely on synthesis and enzymatic synthesis. The chemical method uses cholic acid (CA), chenodeoxycholic acid (CDCA), or hyodeoxycholic acid (HDCA) as starting materials [11]. CA, CDCA, and HDCA are animal-derived raw materials extracted and isolated from bile acids, and their usage poses safety risks owing to the potential contamination with animal pathogens. The UDCA synthesis process also faces challenges such as cost control and process stability [12]. In contrast, plant-derived starting materials can avoid these risks, are widely available, are cost-effective, and have significant potential for industrial application [13].

Wang et al. reported a novel synthetic route for UDCA using 21-hydroxy-20-methylpregn-4-en-3-one [14] (Bisnoralcohol, **BA**) derived from plant sterols through fermentation and side-chain cleavage oxidation [15]. **BA** underwent side-chain selective oxidation, Horner–Wadsworth–Emmons reaction, ethylene glycol protection, allylic oxidation, deprotection reaction, one-pot reduction, and hydrolysis to obtain UDCA in an overall yield of 59%. This route is simple and has high yields, with potential for industrial production and application. In this synthetic route, the selective oxidation of OH-C(20) of BA and the subsequent Horner–Wadsworth–Emmons reaction are key steps that play a crucial role in the extension of the side chain of the steroidal structure.

**Scheme 1:**
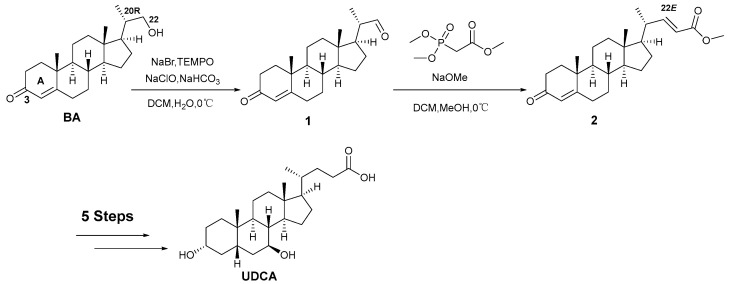
Improved synthetic routes for selective oxidation of OH-C(22) and Horner–Wadsworth–Emmons reaction.

However, there were no reports of cases of reaction scales larger than 10 g, and researchers had not yet investigated potential scale-up effects and impurity formation. In this work, we improved reaction conditions to enhance their suitability for industrial-scale production and successfully scaled up the reaction by a factor of 15; the improved synthetic route was shown in Scheme 1. In the study of selective oxidation of OH-C(22), we used the 2,2,6,6-Tetramethyl-1-piperinedinyloxy (TEMPO)/NaClO/NaBr oxidation system instead of the conventional approach involving tetrabutylammonium bromide (TBAB) and *N*-chlorosuccinimide (NCS). In the study of the Horner–Wadsworth–Emmons reaction, we replaced the conventional triphenylphosphine with a more active trimethyl phosphonoacetate with lower toxicity, leading to milder reaction conditions and lower material costs. Meanwhile, the by-products produced by trimethyl phosphonoacetate were water-soluble phosphates and easy to remove.

Furthermore, we synthesized relevant impurities that may be present in the reaction solution, including the C(22)-carboxylic acid ((17*R*)-3-oxo-10*R*,13*S*-dimethyl-24-cholanoic acid, formed during hydroxyl oxidation)-impurity **1-A**, C(20)-methyl stereoisomeric impurity **2-S** ((20*S*,22*E*)-3-oxo-4,22-choladien-24-oic acid methyl ester) and C(22)-*Z*-ene impurity **2-Z** ((20*R*,22*Z*)-3-oxo-4,22-choladien-24-oic acid methyl ester, formed during THE Horner–Wadsworth–Emmons reaction). We confirmed the origins and generation conditions of these impurities during the scale-up process. Based on these findings, we optimized the process conditions to avoid emulsification. By innovatively adjusting the addition method of reaction materials, we significantly improved the reaction selectivity and yield. These results provided valuable experience and data support for the subsequent industrial application of synthesizing UDCA using **BA** as a raw material.

## 2. Results and Discussion

### 2.1. Study on the Process of the Hydroxyl Oxidation Reaction

**Figure 1:**
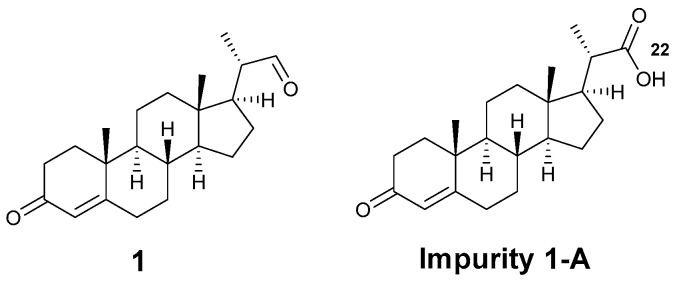
Structural comparison of compound **1** and its carboxylic acid impurity **1-A**.

In the study of selective oxidation of OH-C(22), the following reagents were employed: TEMPO served as the catalyst, NaBr as the co-catalyst, NaClO as the oxidizing agent, and NaHCO_3_ as the buffer. Through this process, the hydroxyl group was successfully converted to an aldehyde group, which led to the selective oxidation of **BA** to obtain compound **1** (Figure 1). When the reaction was scaled up by a factor of 15, scale-up effects were observed, including the emulsification of the reaction mixture and the incomplete conversion of the starting materials. The purified compound **1** with a yield of 89.0% was obtained before the optimization. Considering that NaClO could directly oxidize hydroxyl groups to carboxyls, it was hypothesized that the key impurity, which is the C(22) carboxylic acid **1-A** (Figure 1), might be present in the reaction solution. We synthesized the impurity **1-A** by referring to the literature method [16], using NaClO_2_ as the oxidant, NaH_2_PO_4_ as a buffer, and 2-methyl-2-butene as a scavenger, and confirmed the presence of **1-A** in the reaction system. Since the reaction solution was weakly basic, **1-A** was converted to a sodium salt, which was poorly soluble in both water and dichloromethane(DCM), leading to the emulsification of the reaction mixture and difficulty in separation during subsequent operations.

#### 2.1.1. Effect of NaClO Equivalent

Table 1 illustrated the effect of different amounts of NaClO on the conversion of impurities and target compounds in the reaction. The amount of NaClO directly affected the production of impurity **1-A**, which was thus optimized in this work. We defined the phenomenon, where an intermediate layer remains after cessation of stirring for 15 min, preventing complete separation between the aqueous and organic layers, as emulsification. When 1 equiv. of NaClO was added, 0.85% of the raw **BA** remained unconverted (Table 1, Entry 2). This may be attributed to the partial decomposition of NaClO during the dropwise addition process. When more than 1.1 equiv. of NaClO was added, and the amount of **1-A** increased significantly (Table 1, Entries 4–5), leading to the emulsification of the reaction solution. Therefore, the optimal equivalent dosage during the reaction process was 1.1 equiv. (Table 1, Entry 3).

#### 2.1.2. Effect of Each Reagent in Hydroxyl Oxidation Reaction

The effects of each reagent on the reaction were systematically investigated in Table 2, including TEMPO, NaClO, NaBr, and NaHCO_3_. When all reagents were present, the reaction exhibited excellent selectivity and had high conversion rates of BA (Table 2, Entry 1). It was found that in the absence of TEMPO, the conversion was remarkably reduced, with compound **1** constituting 5.80% of the reaction mixture and impurity **1-A** constituting 0.97% of the reaction mixture (Table 2, Entry 2). In the absence of NaBr, the reaction rate further decreased, and the impurity **1-A** content increased to 2.45% (Table 2, Entry 3). NaBr functioned as a co-catalyst by accelerating the TEMPO-catalyzed oxidation cycle. In addition, NaClO oxidized NaBr to NaBrO, thereby lowering the oxidation potential of NaBr while increasing its selectivity. While in the absence of NaHCO_3_, the time required for the reaction was considerably longer (Table 2, Entry 4). NaHCO_3_ was used to maintain the stability of the pH of the reaction mixture, which is essential for the stability of NaClO and TEMPO.

#### 2.1.3. Effect of Reaction Temperature

Table 3 illustrated how the reaction temperature influenced the content of compound **1** in the reaction solution with temperature. As the temperature increased, the reaction conversion decreased; notably, at 40 °C, over 30% of **BA** remains unreacted (Table 3, Entry 5). Elevated temperatures accelerate the decomposition rate of NaClO, thereby reducing the effective chlorine available for the reaction and compromising the stability of TEMPO. Consequently, the reaction temperature should be controlled within the 0–10 °C range.

### 2.2. Study on the Process of the Horner–Wadsworth–Emmons Reaction

**Figure 2:**
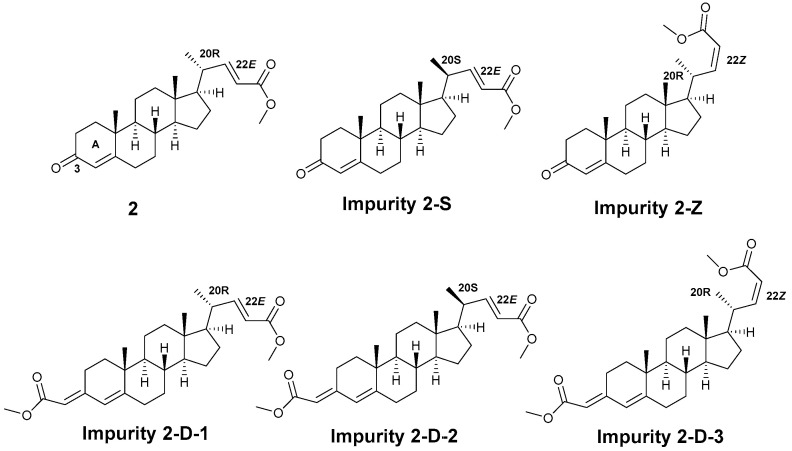
Comparison of the structure of compound **2** and its possible impurities.

In the study of the Horner–Wadsworth–Emmons reaction, a solution containing a mixture of trimethyl phosphonoacetate and compound **1** was introduced into a NaOMe solution to facilitate a nucleophilic addition reaction, resulting in the formation of compound **2**. The purified compound **2** with a yield of 79.1% was obtained before the optimization. The structures of potential impurities were inferred and illustrated in Figure 2 based on the Horner–Wadsworth–Emmons reaction mechanism. (1) The possible presence of chiral isomeric impurities **2-S** with a C(20) methyl orientation different from that of compound **2**. (2) The possible presence of a stereoisomeric impurity **2-Z**, characterized by a *Z*-type configuration at the C(22) double bond, distinguishes it from the *E*-type configuration observed in compound **2**. (3) The possible presence of over-reacted impurities **2-D**, may result from the participation of the carbonyl group at C(3) of the A-ring in compound **2** [17]. The impurities **2-D-1**, **2-D-2,** and **2-D-3** in Figure 2 were three possible isomers in the reaction mixture; no further separation was carried out due to the difficulty of separation and the low content in the mixture.

#### 2.2.1. Synthesis, Characterization, and Validation of Impurities in the Horner–Wadsworth–Emmons Reaction

**Figure 3:**
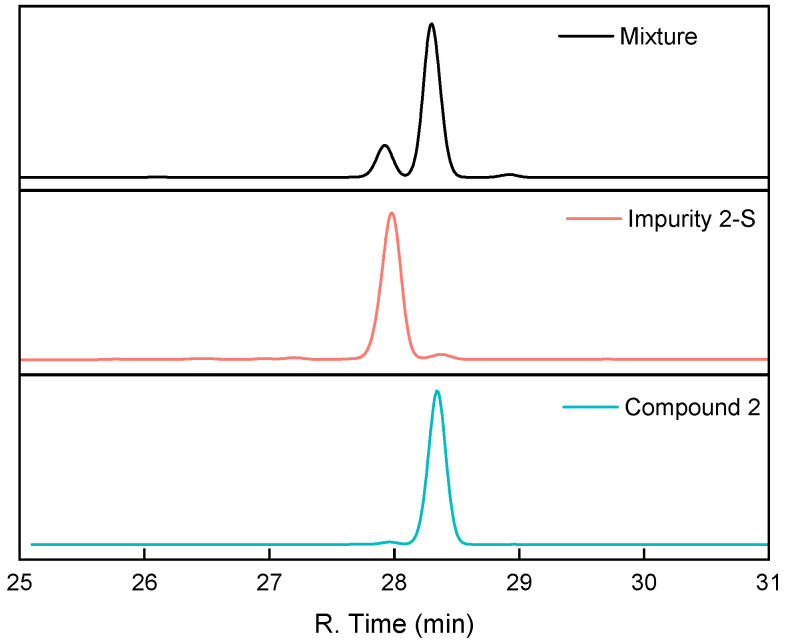
Comparison of peak positions of compound **2** and **2-S** in HPLC.

Compound **1** was refluxed in a mixture of sulfuric acid and ethanol to racemize the C(20) methyl group based on the methods that have been reported [18]. Subsequently, the side chain was extended by the Horner–Wadsworth–Emmons reaction, and the impurity **2-S** (Figure 2) was synthesized successfully and characterized by the ^1^H NMR. The comparison of HPLC peak positions of isolated compounds **2** and **2-S** with the original process reaction mixture was shown in Figure 3.

Further experiments were conducted to verify the generation of **2-S**, which was presumed to be caused by a high concentration of base in the reaction mixture. Compound **1** was stirred with 3 equiv. NaOMe for 3 h, and then trimethyl phosphonoacetate was added. Subsequently, the content of impurity **2-S** in the reaction mixture was compared to the content of compound **2**. The result, which was characterized by HPLC and shown in Entry 3 of Table 4, concluded that the strong base environment also resulted in methyl racemization. Therefore, the high concentration of NaOMe in the reaction was identified as the primary reason for the production of impurity **2-S**.

The reaction mechanism leading to this result might involve the enol tautomerization of compound **1**, which occurs under strongly acidic or basic conditions. This tautomerization results in the formation of a planar intermediate and subsequently the loss of the original chiral center, ultimately causing racemization.

The impurity **2-Z** was obtained from electrophilic bis(2,2,2-trifluoromethyl) phosphonoacetate by using the Still-Gennari modified method of the Horner–Wadsworth–Emmons reaction [19]. Moreover, the structure of impurity **2-Z** was characterized VIA ^1^H NMR.

In addition, the presence of impurity **2-D** and the generation were verified by increasing the equivalents of reagents and reaction time, respectively, and the results monitored by HPLC were shown in Table 4. The content of the impurity **2-D** increased significantly with the addition of higher amounts of trimethyl phosphonoacetate and NaOMe (Table 5, Entries 2–3). Based on HPLC analysis, it was deduced that impurity **2-D** is a compound in which the carbonyl group at C(3) of the A-ring is also involved in the reaction based on compound **2**.

#### 2.2.2. Process Optimization for Impurity **2-S**

In the previous content, it was verified that the origin of **2-S** was a result of the higher concentration of NaOMe compared to compound **1**. In subsequent work, the concentration of NaOMe was reduced in the reaction solution by increasing the amount of solvent. From Table 5, it can be seen that the content of impurity **2-S** was greatly reduced under a high solvent dosage. However, it led to lower productivity and higher costs (Table 6, Entry 3) as the weight ratio of solvent to compound **1** exceeded 30:1, thus a new way of adding reagents was applied. In the improved experimental method, NaOMe was first dissolved in methanol (0.5 M) and mixed with trimethyl phosphonoacetate (1.2 equiv.) to form a solution under nitrogen. Subsequently, this solution was added dropwise to a DCM solution of compound **1** at −10 °C. In this way, the NaOMe in the reaction mixture was consumed promptly as the titration proceeded so that the concentration of NaOMe in the reaction mixture could be kept low until the end of the reaction. The results showed that the content of **2-S** was effectively reduced, and the yield of the whole reaction was increased to 90.8% (Table 6, Entry 4).

## 3. Materials and Methods

General Chemistry Methods: All commercially available materials and solvents were used directly without further purification. The reactions were monitored using a Shimadzu LC-20AT HPLC instrument (Kyoto, Japan). The ^1^H NMR and ^13^C NMR spectra were recorded using a Bruker Advance 400 MHz nuclear magnetic resonance (NMR) spectrometer (Billerica, MA, USA) with (CH_3_)_4_Si (TMS) as an internal standard. Mass spectrometry results were performed on a high-performance electrospray-quadrupole-time-of-flight LC/MS/MS tandem mass spectrometer, microTOF-Q II, from Bruker Daltonics (Billerica, MA, USA).

### 3.1. Synthesis of (20R)-3-Oxopregna-4-en-22-al (**1**)

Compound **BA** (150.1 g, 454 mmol), TEMPO (1.4 g, 9 mmol), DCM (563.6 mL), NaBr (4.8 g, 46 mmol), NaHCO_3_ (11.5 g, 137 mmol), and deionized water (160.7 mL) were added sequentially. The reaction mixture was carried out at 0–5 °C. A 12% aqueous solution of NaClO (256.0 mL, 454 mmol) was slowly added dropwise over 30 min while maintaining the temperature within the range of 0–5 °C. After the addition was complete, the mixture was stirred for 30 min. Completion of the reaction was confirmed using HPLC analysis, with a substrate content of less than 0.5%. Na_2_S_2_O_3_ (7.0 g, 28 mmol) was added, and the mixture was stirred at 0–5 °C for 30 min. The mixture was allowed to stand, and the aqueous layer was extracted with DCM (110.0 mL × 3). The organic phases were combined and washed with saturated Na_2_CO_3_ solution (100.0 mL × 2). The organic phase was dried over with Na_2_SO_4_ and concentrated under reduced pressure in a 50 °C water bath (vacuum: 0.09 MPa) to yield 186.7 g of a light-reddish solid. The solid was redispersed in MeOH (150.0 mL) and stirred at 0 °C for 2 h. The mixture was filtered under reduced pressure, and the filter cake was washed with MeOH (125.0 mL) at 0 °C. The filter cake was dried in an oven at 50 °C for 24 h to afford 134.0 g of an off-white powder with a yield of 95.2% and a purity of 98.12%. ^1^H NMR (400 MHz, DMSO-*d*_6_) δ 9.53 (d, *J* = 2.9 Hz, 1H), 5.62 (s, 1H), 2.47–2.32 (m, 3H), 2.19 (ddt, *J* = 35.7, 16.7, 3.5 Hz, 2H), 1.94 (ddt, *J* = 22.2, 12.7, 3.3 Hz, 2H), 1.78 (dq, *J* = 12.9, 5.1, 4.5 Hz, 2H), 1.64–1.36 (m, 7H), 1.20 (dd, *J* = 12.5, 4.3 Hz, 2H), 1.15 (s, 3H), 1.03 (d, *J* = 6.8 Hz, 3H), 0.93 (dddd, *J* = 22.9, 15.1, 9.4, 4.9 Hz, 3H), 0.72 (s, 3H). ^13^C NMR (101 MHz, DMSO-*d*_6_) δ 205.26, 197.98, 170.91, 123.14, 54.55, 53.15, 50.45, 48.62, 42.47, 38.66, 38.12, 35.10, 34.89, 33.59, 31.96, 31.64, 26.45, 24.12, 20.52, 16.87, 13.10, 11.98. ESI-MS, *m/z* [M+H]^+^ 328.24, found: 328.25.

### 3.2. Synthesis of (20R,22E)-3-Oxopregna-4,22-dien-24-oic Acid Methyl Ester (**2**)

Compound **1** (130.1 g, 396 mmol) was added to DCM (150.0 mL) and stirred at 0 °C. Trimethyl phosphonoacetate (69.0 mL, 433 mmol) was dissolved in a NaOMe solution (20% *w*/*w*, 120.0 mL, 425 mmol) to prepare a mixed solution, which was added dropwise to the flask over 30 min. The reaction mixture was stirred at 0–5 °C for 1 h. Completion of the reaction was confirmed using HPLC analysis, with a substrate content of less than 0.5%. Hydrochloric acid (36% *w*/*w*, 3.0 mL, 36 mmol) was added dropwise at 0–5 °C until the pH reached 3. Water (200.0 mL) was then added, and the mixture was stirred at 0–5 °C for 30 min. The mixture was allowed to stand, and the aqueous phase was extracted with DCM (75.0 mL × 2). All organic layers were combined and washed with saturated sodium carbonate solution (100.0 mL × 2). The organic phase was dried over Na_2_SO_4_ and concentrated under reduced pressure in a 50 °C water bath (vacuum: 0.09 MPa), yielding 162.1 g of a pale yellow solid. MeOH (110.0 mL) was added, and the mixture was stirred at 0 °C for 2 h. The resulting mixture was filtered under reduced pressure, and the filter cake was washed with MeOH (90.0 mL) at 0 °C. The filter cake was dried in an oven at 50 °C for 24 h, affording 120.4 g of a white powder in a yield of 90.8% with a purity of 97.80%. ^1^H NMR (400 MHz, DMSO-*d*_6_) δ 6.74 (dd, *J* = 15.6, 9.0 Hz, 1H), 5.81 (d, *J* = 15.6 Hz, 1H), 5.62 (s, 1H), 3.63 (s, 3H), 2.45–2.10 (m, 5H), 1.96 (d, *J* = 12.4 Hz, 2H), 1.76 (d, *J* = 12.7 Hz, 1H), 1.65–1.32 (m, 6H), 1.22 (td, *J* = 10.6, 3.6 Hz, 3H), 1.14 (s, 3H), 1.08 (s, 1H), 1.04 (d, *J* = 6.5 Hz, 3H), 1.01–0.84 (m, 3H), 0.72 (s, 3H). ^13^C NMR (101 MHz, DMSO-*d*_6_) δ 197.98, 170.96, 166.41, 154.66, 123.12, 118.48, 55.11, 54.07, 53.15, 51.16, 42.25,39.73, 39.31, 38.12, 35.10, 34.90, 33.58, 31.98, 31.62, 27.58, 23.78, 20.54, 18.95, 16.87, 11.95. ESI-MS, *m/z* [M+H]^+^ 385.23, found: 385.25.

### 3.3. Synthesis of (20R)-3-Oxopregna-4-en-22-oic Acid (**1-A**)

Compound **1** (3.12 g, 9.4 mmol), tert-butanol (10.00 mL), and 2-methyl-2-butene (46.00 mL) were added to a 250 mL three-necked flask. NaH_2_PO_4_ (8.00 g, 66.7 mmol) and NaClO_2_ (2.05 g, 23.4 mmol) were dissolved in water (50.0 mL) and added dropwise to the three-necked flask over 15 min. The mixture was stirred at 0 °C for 1 h. Na_2_SO_3_ (2.79 g, 22.2 mmol) was added, and the mixture was stirred for 15 min to quench the reaction. The reaction mixture was concentrated under reduced pressure in a 40 °C water bath (vacuum: 0.09 MPa). Ethyl acetate (110.0 mL) was added, and the pH was adjusted to 1 using hydrochloric acid (*w* = 36%, 3.01 mL, 36 mmol). The mixture was allowed to stand, and the aqueous phase was extracted with ethyl acetate (30.00 mL × 3). The combined organic phases were mixed with water (300.00 mL), and Na_2_CO_3_ (1.50 g, 14.1 mmol) was added to adjust the pH to 10. The mixture was stirred for 30 min. The mixture was allowed to stand, and hydrochloric acid (*w* = 36%, 1.87 mL, 20.7 mmol) was added to the aqueous phase to adjust the pH to 1. A white suspension formed, which was filtered under reduced pressure and dried in an oven at 50 °C for 24 h, affording 0.31 g of white solid in a yield of 9.47%. ^1^H NMR (400 MHz, DMSO-*d*_6_) δ 11.92 (s, 1H), 5.62 (d, *J* = 1.7 Hz, 1H), 2.39 (ddd, *J* = 16.5, 14.7, 4.9 Hz, 2H), 2.27–2.12 (m, 3H), 1.94 (ddt, *J* = 22.3, 12.3, 3.2 Hz, 2H), 1.76 (dt, *J* = 13.0, 2.9 Hz, 1H), 1.71–1.41 (m, 7H), 1.30 (dddd, *J* = 44.6, 22.6, 12.9, 4.1 Hz, 3H), 1.14 (s, 3H), 1.10 (d, *J* = 6.8 Hz, 3H), 0.92 (dtd, *J* = 15.0, 12.0, 4.0 Hz, 2H), 0.69 (s, 3H). ^13^C NMR (101 MHz, DMSO-*d*_6_) δ 205.26, 197.98, 170.91, 123.14, 54.55, 53.15, 50.45, 48.62, 42.47, 38.66, 38.12, 35.10, 34.89, 33.59, 31.96, 31.64, 26.45, 24.12, 20.52, 16.87, 13.10, 11.98. ESI-MS, *m/z* [M+H]^+^, 344.28, found: 343.05.

### 3.4. Synthesis of (20S,22E)-3-Oxopregna-4,22-dien-24-oic Acid Methyl Ester (**2-S**)

Compound **1** (6.70 g, 20.4 mmol), anhydrous ethanol (320.0 mL), and H_2_SO_4_ (*w* = 50%, 128.0 mL) were added to a 1 L single-necked flask equipped with a reflux condenser and heated under reflux for 30 min. After the reaction, the mixture was cooled in an ice-water bath and extracted with methyl tert-butyl ether (200.0 mL × 2). The organic phase was separated and washed successively with water (100.00 mL) and saturated brine (83.00 mL). It was dried over Na_2_SO_4_ and concentrated under reduced pressure in a 40 °C water bath (vacuum: 0.09 MPa), yielding a white residue. The residue and DCM (115.00 mL) were transferred to a 500 mL single-necked flask. Trimethyl phosphonoacetate (4.13 g, 22.7 mmol) and NaOMe (1.25 g, 23.1 mmol) were added, and the mixture was stirred for 1 h. Hydrochloric acid (*w* = 36%) was added dropwise at 0–5 °C to adjust the pH to 1. Water (50.00 mL) was added, and the mixture was stirred at 0–5 °C for 30 min. The mixture was allowed to stand, and the aqueous phase was extracted with DCM (25.00 mL × 2). The combined organic phases were washed with saturated Na_2_CO_3_ solution (30.00 mL × 2). The organic phase was dried over anhydrous Na_2_SO_4_ and concentrated under reduced pressure in a 50 °C water bath (vacuum: 0.09 MPa), yielding a pale yellow solid. The product was purified using silica gel column chromatography (eluent: ethyl acetate/petroleum ether = 1:4) repeated four times to obtain compound **2-S** (0.41 g, yield: 5.13%). ^1^H NMR (400 MHz, DMSO-*d*_6_) δ 6.79 (dd, *J* = 15.6, 9.9 Hz, 1H), 5.84 (d, *J* = 15.6 Hz, 1H), 5.62 (d, *J* = 1.6 Hz, 1H), 3.64 (s, 3H), 2.43–2.11 (m, 5H), 1.94 (ddd, *J* = 13.3, 5.1, 3.1 Hz, 1H), 1.87–1.74 (m, 2H), 1.68–1.38 (m, 6H), 1.33–1.21 (m, 4H), 1.12 (s, 3H), 0.94 (d, *J* = 6.6 Hz, 3H), 0.91–0.78 (m, 3H), 0.65 (s, 3H). ESI-MS, *m/z* [M+H]^+^, 385.23, found: 385.25.

### 3.5. Synthesis of (20R,22Z)-3-Oxopregna-4,22-dien-24-oic Acid Methyl Ester (**2-Z**)

Bis(2,2,2-trifluoroethyl) phosphorylacetic acid methyl ester (0.84 g, 2.6 mmol), 18-crown-6 ether (0.35 g, 1.3 mmol), anhydrous tetrahydrofuran (20.00 mL), and 0.5 M potassium bis(trimethylsilyl)amide in toluene (5.00 mL, 2.5 mmol) were added to a 25 mL single-necked flask and stirred at −78 °C. Compound **1** (0.40 g, 1.2 mmol) was dissolved in 5.0 mL of tetrahydrofuran and added dropwise under a nitrogen atmosphere. The mixture was stirred at −78 °C for 3 h. Saturated ammonium chloride solution (20.00 mL) was added at room temperature and stirred for 10 min. DCM (25.00 mL) was then added, and the mixture was allowed to settle. The organic phase was washed twice with water (20.00 mL × 2) and saturated brine (20.00 mL × 2), dried over anhydrous Na_2_SO_4_ and concentrated under reduced pressure in a 40 °C water bath (vacuum: 0.09 MPa), yielding a white residue. The product was purified via silica gel column chromatography (eluent: ethyl acetate/petroleum ether = 1:4), yielding compound **2-*Z*** as a white solid (0.12 g, yield: 32.52%). ^1^H NMR (400 MHz, DMSO-*d*_6_) δ 6.07 (t, *J* = 11.0 Hz, 1H), 5.67 (d, *J* = 11.5 Hz, 1H), 5.62 (s, 1H), 3.62 (s, 3H), 2.44–2.34 (m, 2H), 2.19 (dd, *J* = 32.0, 15.6 Hz, 2H), 1.96 (d, *J* = 12.2 Hz, 2H), 1.76 (d, *J* = 13.1 Hz, 1H), 1.55 (dt, *J* = 24.5, 11.2 Hz, 5H), 1.43–1.31 (m, 2H), 1.22 (d, *J* = 11.3 Hz, 3H), 1.15 (s, 3H), 0.98 (d, *J* = 6.6 Hz, 3H), 0.93–0.85 (m, 3H), 0.73 (s, 3H). ^13^C NMR (101 MHz, DMSO-*d*_6_) δ 198.01, 170.14, 165.83, 155.85, 123.12, 116.13, 55.26, 54.64, 53.19, 50.85, 42.17, 39.94, 39.73, 38.14, 34.91, 34.23, 33.60, 31.98, 31.64, 27.67, 24.04, 20.57, 19.30, 16.88, 12.15. ESI-MS, *m/z* [M+H]^+^, 385.23, found: 385.25.

## 4. Conclusions

In this work, we investigated the selective hydroxyl oxidation and Horner–Wadsworth–Emmons reaction involved in synthesizing UDCA from the plant-derived feedstock **BA**, revealing significant scale-up effects that were subsequently optimized. In hydroxyl oxidation, a C(22) carboxylic acid impurity was synthesized. By employing more cost-effective reagents and optimizing the process to resolve emulsification issues, the yield was improved from 89.0% to 95.2%. In the Horner–Wadsworth–Emmons reaction, C(20) methyl isomer impurity and a C(22)-*Z*-ene isomer impurity were synthesized and characterized. It was determined that the C(20)-methyl racemate was the key factor affecting the reaction yield. By modifying the feeding strategy and adjusting the reagent concentration, the yield increased from 79.1% to 90.8%. Overall, the two-step process yield was enhanced to 86.5%.

## Figures and Tables

**Table 1:** Effect of NaClO equivalent on the composition of the reaction mixture.

Entry	NaClO (Equiv.)	BA (HPLC Area%)	1-A (HPLC Area%)	Emulsification
1	0.9	2.69	0.12	NO
2	1.0	0.85	0.23	NO
3	1.1	0.33	0.31	NO
4	1.2	0.12	0.86	YES
5	1.4	0.12	1.20	YES

**Table 2:** Effects of each reagent on reaction time and composition of the reaction mixture.

Entry	Material Addition Condition	Time	BA (HPLC Area%)	Compound 1 (HPLC Area%)	Impurity 1-A (HPLC Area%)
1	Complete material addition	0.5 h	0.21	97.12	0.13
2	TEMPO was not added	12 h	60.99	5.80	0.97
3	NaBr was not added	3 h	0.37	95.04	2.45
4	NaHCO_3_ was not added	24 h	44.27	53.07	0.09

**Table 3:** Effect of reaction temperature on the conversion of the hydroxyl oxidation reaction.

Entry	Temp.	BA (HPLC Area%)	Compound 1 (HPLC Area%)
1	0 °C	0.11	97.12
2	10 °C	0.12	97.23
3	20 °C	0.18	96.02
4	30 °C	0.16	95.96
5	40 °C	32.83	65.40

**Table 4:** The contents of impurities produced by the Horner–Wadsworth–Emmons reaction after two racemic methods were compared.

Entry	Treatment of Racemization	Compound 2 (HPLC Area%)	Impurity 2-S (HPLC Area%)
1	None	82.51	13.64
2	Strong acid ^1^	41.39	39.20
3	Strong base ^2^	51.37	42.56

^1^ Compound 1 was refluxed in a mixed solution of 50% sulfuric acid and methanol for 1 h and then subjected to the Horner–Wadsworth–Emmons reaction. ^2^ Compound 1 was stirred in 3 equiv. of NaOMe for 3 h then subjected to the Horner–Wadsworth–Emmons reaction.

**Table 5:** Comparison of impurity content after two racemization methods.

Entry	Compound 1:NaOMe:Trimethyl Phosphonoacetate (Equiv.)	Time	Impurity 2-D (HPLC Area%)
1	1:1.05:1.05	1 h	0.65
2	1:1.05:1.05	5 h	1.80
3	1:3:3	5 h	43.83

**Table 6:** Comparison of main impurity contents under different solvent dosages and NaOMe-feeding methods.

Entry	DCM:1 ^1^	NaOMe ^2^	Compound 2 (HPLC Area%)	Impurity 2-S (HPLC Area%)	Impurity 2-Z (HPLC Area%)	Impurity 2-D (HPLC Area%)	Yield
1	5	Solid, Add all at once	82.51	13.64	1.21	0.65	79.1%
2	10	Solid, Add all at once	84.53	9.86	1.11	0.18	82.5%
3	30	Solid, Add all at once	95.38	2.85	0.69	0.14	91.5%
4	5	Methanol solution (*w* = 20%)	96.64	0.85	1.03	0.19	90.8%

^1^ This represented the weight ratio of DCM to compound **1**. ^2^ This represented the feeding method of NaOMe.

## Data Availability

The data underlying this study are available in the published article. and its online Supplementary Materials.

## Supplementary Materials

The following supporting information can be downloaded at https://www.mdpi.com/article/10.3390/molecules30071454/s1: Page S1: The route developed by Wang et al. to synthesize UDCA from BA mentioned in the article; Page S1:Materials and instruments; Page S1–S5: HPLC Detection Method and spectra; Pages S6–S10: ^1^H NMR and ^13^C NMR spectra; Pages S10–S11: Mass analysis and LC-MS results for all the compounds and impurities.
