# Supplementary material for: Studies on the Synthesis Process of Plant-Derived Ursodeoxycholic Acid Intermediates
Source: Molecules. 2025 Mar 25;30(7):1454. doi: 10.3390/molecules30071454 (PMC11990328; doi:10.3390/molecules30071454)
Supplement: Supplementary file 1 [file molecules-30-01454-s001.zip › molecules-3537092-supplementary.pdf]

Supplementary Materials

Studies on the synthesis process of plant-derived ursodeoxycholic acid intermediates

Shaoxiong Jing [1], Zhongyue Wang [1], Yuan Wang [1], Yingquan Yang [2], Jian Song [1,*], and Bao Zhang[1,*]

1. School of Chemical Engineering and Technology, Tianjin University, Tianjin, 300350, China.

2. Suzhou Entai New Materials Technology Company, Suzhou, 215000, China.

* Correspondence author:

E-mail address: baozhang@tju.edu.cn. (B.Z.)

Contents:

The route developed by Wang et al. to synthesize UDCA from BA S**1**

Materials and instruments S**1**

HPLC Detection Method S**1**

^{1}H NMR and ^{13}C NMR spectra of compound **1** S**6**

^{1}H NMR and ^{13}C NMR spectra of compound **2** S**7**

^{1}H NMR and ^{13}C NMR spectra of impurity **1-A** S**8**

^{1}H NMR spectra of impurity **2-S** S**9**

^{1}H NMR and ^{13}C NMR spectra of impurity **2-Z** S**10**

Mass analysis and LC-MS results for all the compounds and impurities S**11**

1. The route developed by Wang et al. to synthesize UDCA from BA mentioned in the article [15]

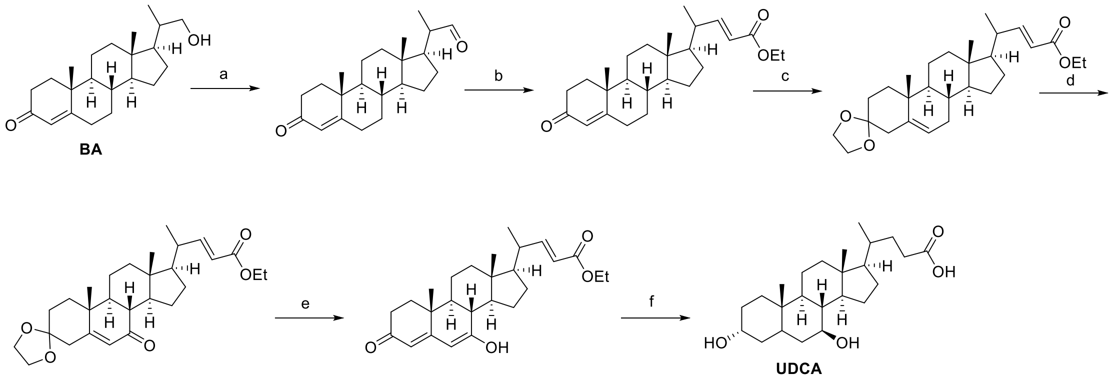

Reagents and conditions: (a) TEMPO, $NaHCO_3$, TBAB, NCS, CH_2Cl_2, H_2O, 0 °C, 95%; (b) Ph_3 = $CHCOOC_2H_5$, toluene, reflux, 98%;(c) ethylene glycol, pTSA, benzene, reflux, 88%; (d) PDC, NHPI, acetone, H_2O, rt, 85%; (e) H_2SO_4, THF, rt, 98%; (f) RaneyNi, H_2, t-BuONa, 2-methyl tetrahydrofuran, i-PrOH, 90 °C, 87%

Scheme S1 The route developed by Wang et al. to synthesize UDCA from BA

2. Materials and instruments

BA(Bisnoralcohol) was purchased from Shandong Sito Biotechnology Co. Ltd and The company's website was as follows: https://www.sitobiotech.com/. Dichloromethane, methanol, ethanol, ethyl acetate, tert-butanol, and petroleum ether were purchased from Shanghai Titan Technology Co.; Unless otherwise noted, all commercial materials were used directly without further purification.

Our reactors primarily consist of glassware from Shuniu, including single-neck round-bottom flasks (25mL, 100mL, 250mL, 500mL) and 500mL three-neck flasks. Heating/stirring equipment includes Gongyi Jinghua's DF-101S collector-type thermostatic magnetic stirrer and DFY-30 low-temperature thermostatic reaction bath.

The reactions were monitored by a Shimadzu LC-20AT HPLC instrument. The ^{1}H NMR and ^{13}C NMR spectra were recorded using a Bruker Advance 400 MHz nuclear magnetic resonance (NMR) spectrometer with TMS as an internal standard. The mass spectra were recorded on a Bruker MicrOTOF-Q II.

3. HPLC Detection Method

High-performance liquid chromatography (HPLC) served as the primary analytical method, employing a Shimadzu LC-2050 system (Japan). Chromatographic separation was achieved using an Agilent Poroshell 120 SB-C8 column (4.6 mm × 150 mm, 2.7 μm particle size).

Test solutions were prepared by accurately weighing appropriate quantities of the sample, followed by ultrasonic dissolution in acetonitrile and dilution to 0.3 mg/mL. Analyses were conducted in accordance with the Chinese Pharmacopoeia (2020 Edition, General Principles 0512 under Part IV).

Chromatographic conditions were as follows:

- Stationary phase: Octadecylsilane-bonded silica gel
- Mobile phases: A (water), B (acetonitrile) with gradient elution programmed as specified in Table Table S1
- Detection: UV absorbance at 254 nm
- Flow rate: 0.6 mL/min
- Column temperature: 40 °C
- Injection volume: 10 μL

Impurity quantitation was performed using area normalization method.

Table S1 HPLC detection gradient

Time	Mobile phase A(%)	Mobile phase B(%)
0	60	40
40	0	100
45	0	100
45.1	60	40
50	60	40

The HPLC spectra of the relevant compounds as well as the reaction mixture are shown below: (The meaning of the “峰表” mentioned in the figure is “Peak Table”, and the meaning of the “检测器” mentioned in the figure is “Detectors for high--performance liquid chromatography”)

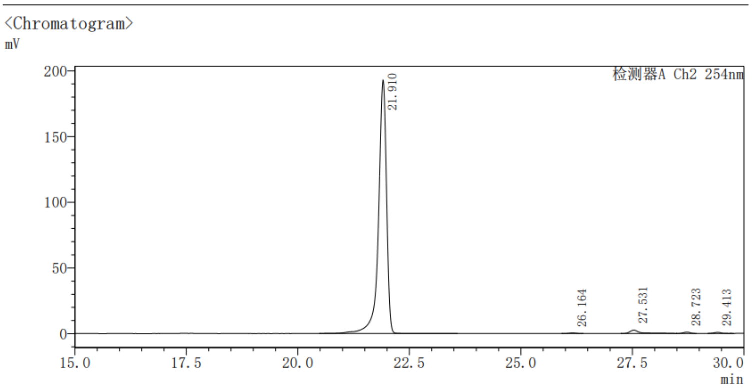

<峰表>
检测器A Ch2 254nm

Peak#	Ret. time	Area	Area%	Height	Compound ID
1	14.215	7984	0.316	537	
2	14.696	26053	1.030	1356	
3	21.910	2427877	96.006	192578	BA-1
4	26.164	4463	0.176	427	
5	27.531	42378	1.676	2536	
6	28.723	10655	0.421	1023	
7	29.413	9467	0.374	824	
总计		2528877	100.000	199281	

Figure S1 HPLC spectrum of **Compound 1**

<Chromatogram>

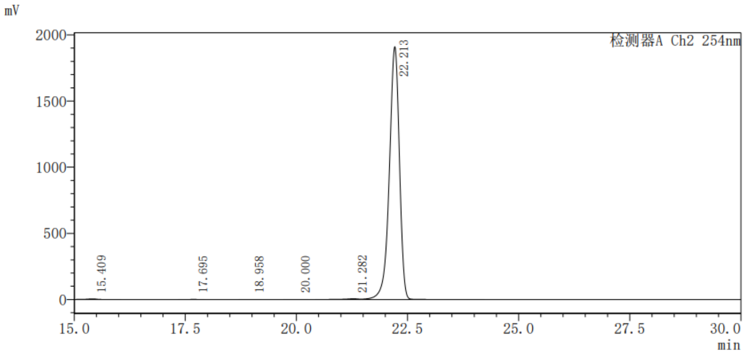

<峰表>

检测器A Ch2 254nm

Peak#	Ret. time	Area	Area%	Height	Compound ID
1	8.163	39915	0.131	3888	
2	14.137	43697	0.143	1252	
3	15.409	69943	0.230	4454	BA-1-A
4	17.695	6065	0.020	319	
5	18.958	3664	0.012	212	
6	20.000	3052	0.010	115	
7	21.282	108591	0.356	5390	
8	22.213	30149606	98.953	1909747	BA-1
9	33.613	19585	0.064	1449	
10	41.981	24601	0.081	1821	
总计		30468721	100.000	1928647	

Figure S2 HPLC spectrum of reaction mixture of **compound 1**

<Chromatogram>

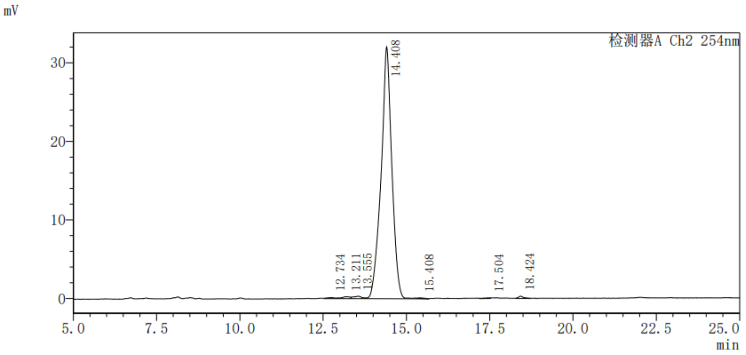

<峰表>

检测器A Ch2 254nm

Peak#	Ret. time	Area	Area%	Height	Compound ID
1	12.734	2361	0.319	143	
2	13.211	4385	0.593	240	
3	13.555	5449	0.737	310	
4	14.408	721923	97.607	32042	BA-1-A
5	15.408	1103	0.149	88	
6	17.504	1304	0.176	89	
7	18.424	3096	0.419	269	
总计		739622	100.000	33181	

Figure S3 HPLC spectrum of **impurity 1-A**

<Chromatogram>

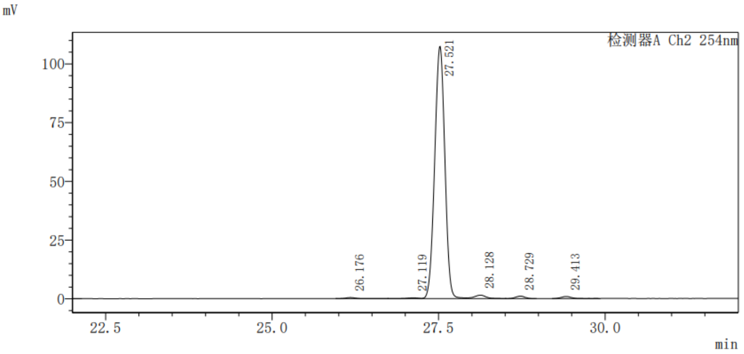

<峰表>

检测器A Ch2 254nm

Peak#	Ret. time	Area	Area%	Height	Compound ID
1	21.950	2349	0.197	155	
2	26.176	5111	0.429	472	
3	27.119	4240	0.356	352	BA-2-S
4	27.521	1140565	95.657	107337	BA-2
5	28.128	18853	1.581	1456	BA-2-Z
6	28.729	10691	0.897	1061	
7	29.413	9521	0.798	868	
8	35.210	1020	0.086	66	
总计		1192349	100.000	111768	

Figure S4 HPLC spectrum of **compound 2**

<Chromatogram>

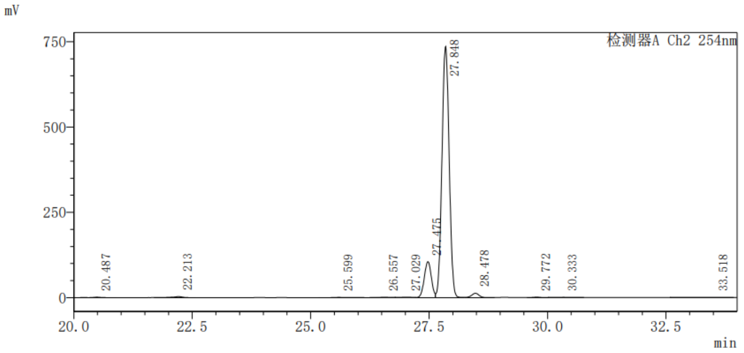

<峰表>

检测器A Ch2 254nm

Peak#	Ret. time	Area	Area%	Height	Compound ID
1	17.455	74746	0.811	7768	
2	20.487	13244	0.144	1221	
3	22.213	42789	0.464	2953	
4	25.599	10697	0.116	618	
5	26.557	9750	0.106	825	
6	27.029	9984	0.108	866	
7	27.475	1093198	11.860	105446	BA-2-S
8	27.848	7792288	84.539	735576	BA-2
9	28.478	140389	1.523	12597	BA-2-Z
10	29.772	15413	0.167	1310	
11	30.333	5613	0.061	497	
12	33.518	9230	0.100	459	BA-2-D
总计		9217343	100.000	870137	

Figure S5 HPLC spectrum of reaction mixture of **compound 2**

<Chromatogram>

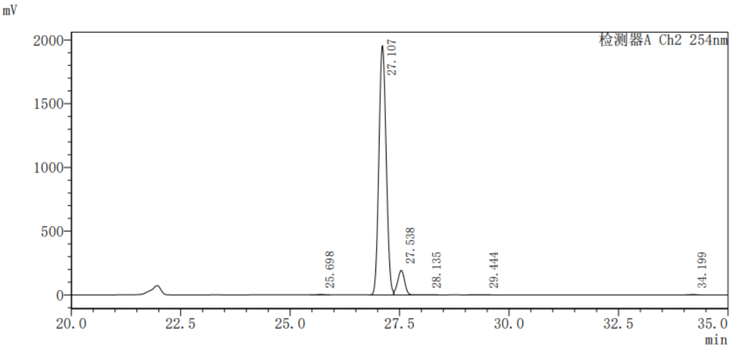

<峰表>

检测器A Ch2 254nm

Peak#	Ret. time	Area	Area%	Height	Compound ID
1	25.698	39839	0.160	3025	
2	27.107	22648765	91.048	1951662	BA-2-S
3	27.538	2129095	8.559	192264	BA-2
4	28.135	18033	0.072	1051	BA-2-Z
5	29.444	12439	0.050	834	
6	34.199	27462	0.110	2895	
总计		24875632	100.000	2151732	

Figure S6 HPLC spectrum of **impurity 2-S**

<Chromatogram>

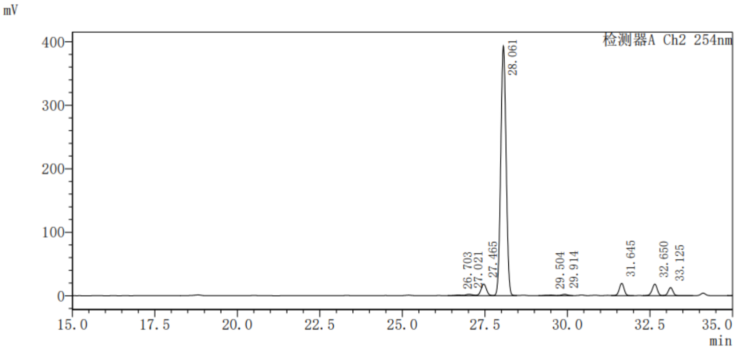

<峰表>

检测器A Ch2 254nm

Peak#	Ret. time	Area	Area%	Height	Compound ID
1	26.703	14190	0.266	1020	
2	27.021	31590	0.593	2158	BA-2-S
3	27.465	212139	3.981	18228	BA-2
4	28.061	4456765	83.644	392987	BA-2-Z
5	29.504	14904	0.280	804	
6	29.914	25883	0.486	2037	
7	31.645	200026	3.754	19380	
8	32.650	197299	3.703	18056	
9	33.125	126336	2.371	12653	
10	35.151	49142	0.922	5382	
总计		5328275	100.000	472707	

Figure S7 HPLC spectrum of **impurity 2-Z**

<Chromatogram>

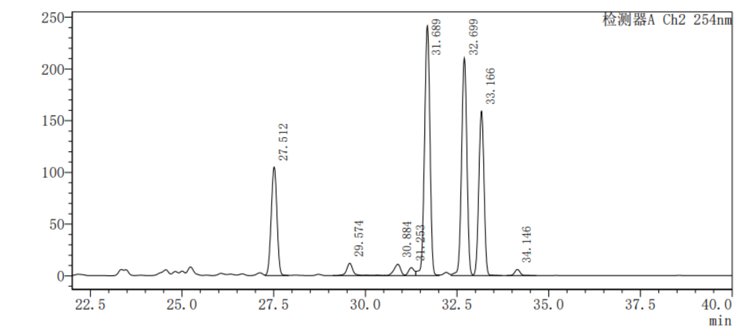

<峰表>

检测器A Ch2 254nm

Peak#	Ret. time	Area	Area%	Height	Compound ID
1	27.512	1152827	14.846	104960	BA-2
2	29.574	144764	1.864	11980	
3	30.884	137893	1.776	10932	
4	31.253	83455	1.075	7684	
5	31.689	2477044	31.899	241347	
6	32.699	2125268	27.369	210120	
7	33.166	1582579	20.380	158654	
8	34.146	61344	0.790	5912	
总计		7765174	100.000	751590	

Figure S8 HPLC spectrum of **impurity 2-D**

4. ^{1}H NMR and ^{13}C NMR spectra of compound 1

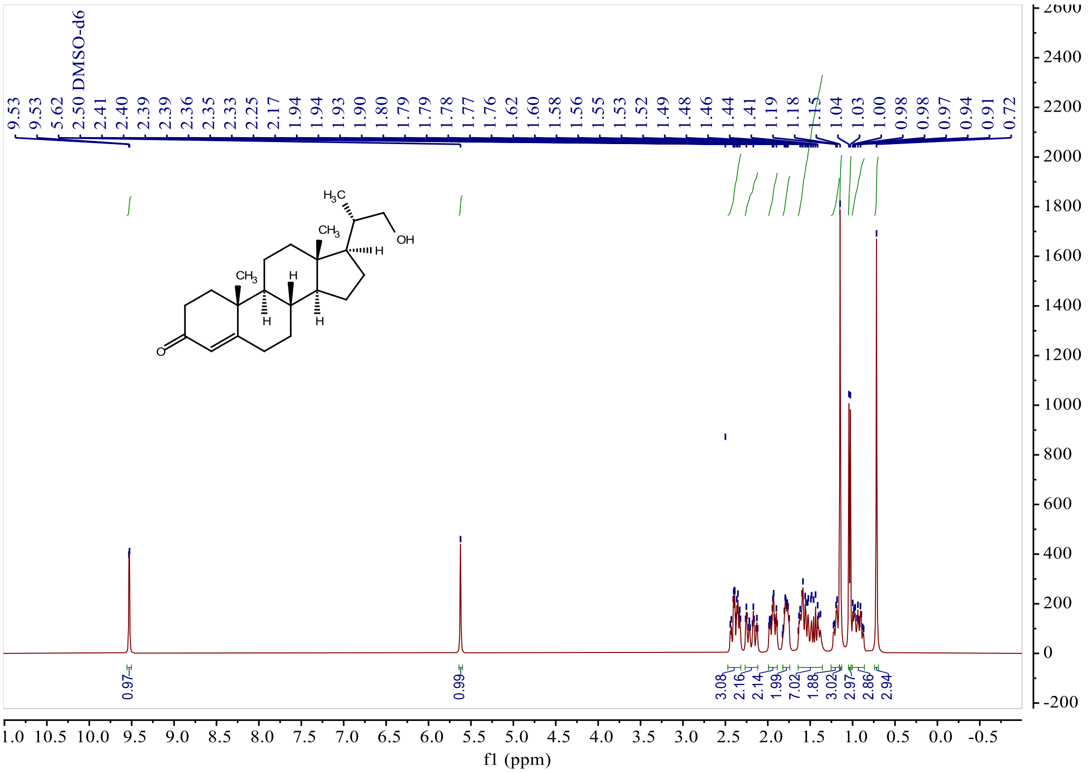

Figure S9 ^{1}H NMR (400 MHz, DMSO-d6) spectrum of **compound 1**

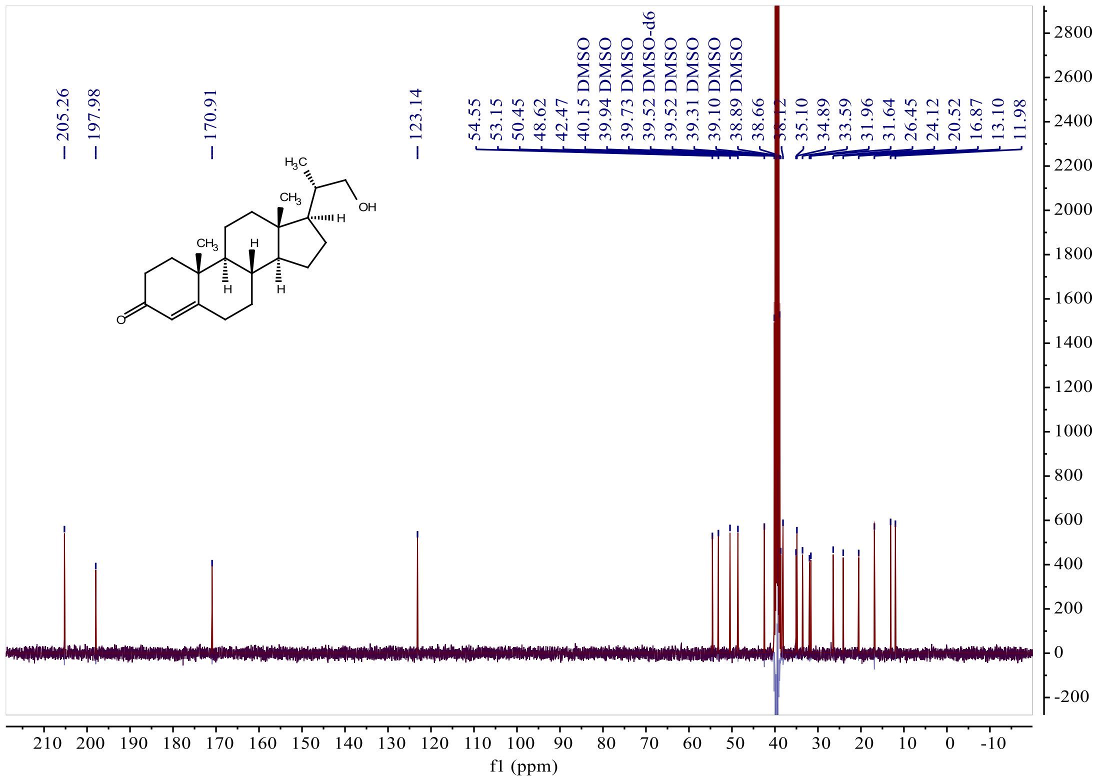

Figure S10 ^{13}C NMR (101 MHz, DMSO-d6) spectrum of **compound 1**

5. ^{1}H NMR and ^{13}C NMR spectra of compound 2

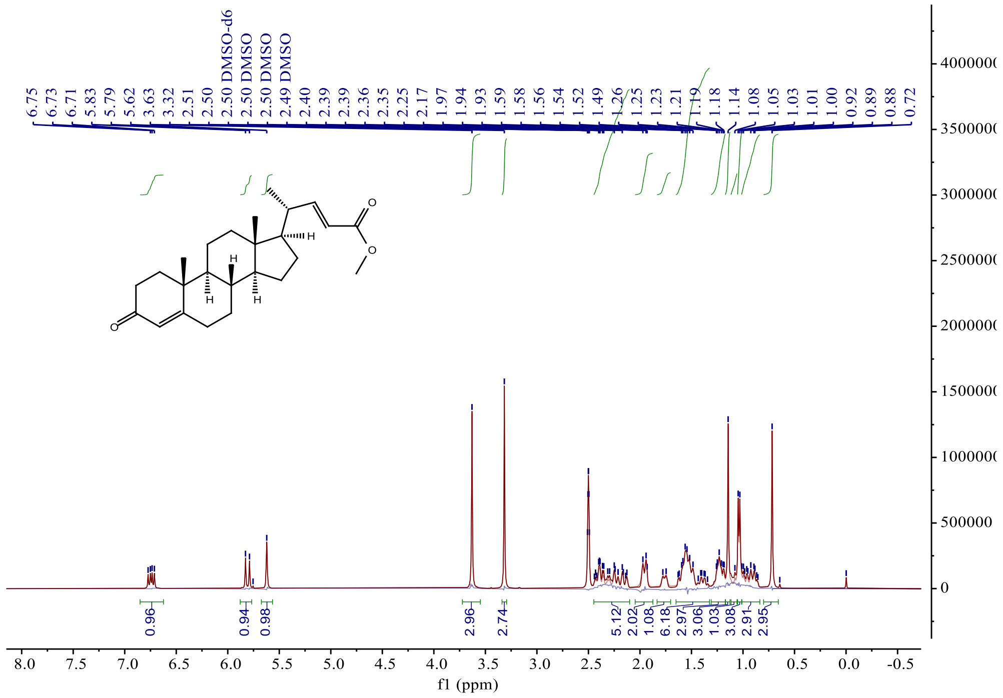

Figure S11 ^{1}H NMR (400 MHz, DMSO-d6) spectrum of **compound 2**

^{13}C NMR (101 MHz, DMSO-d_6) of compound 2

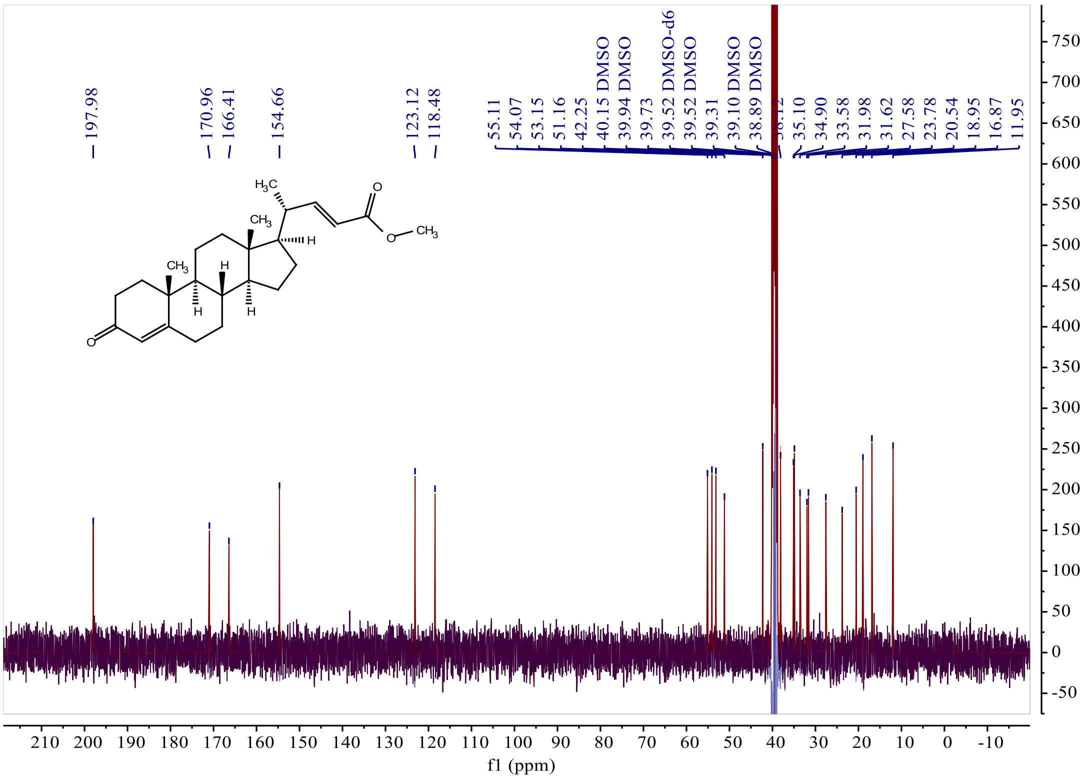

Figure S12 ^{13}C NMR (101 MHz, DMSO-d6) spectrum of **compound 2**

6. ^{1}H NMR and ^{13}C NMR spectra of impurity 1-A

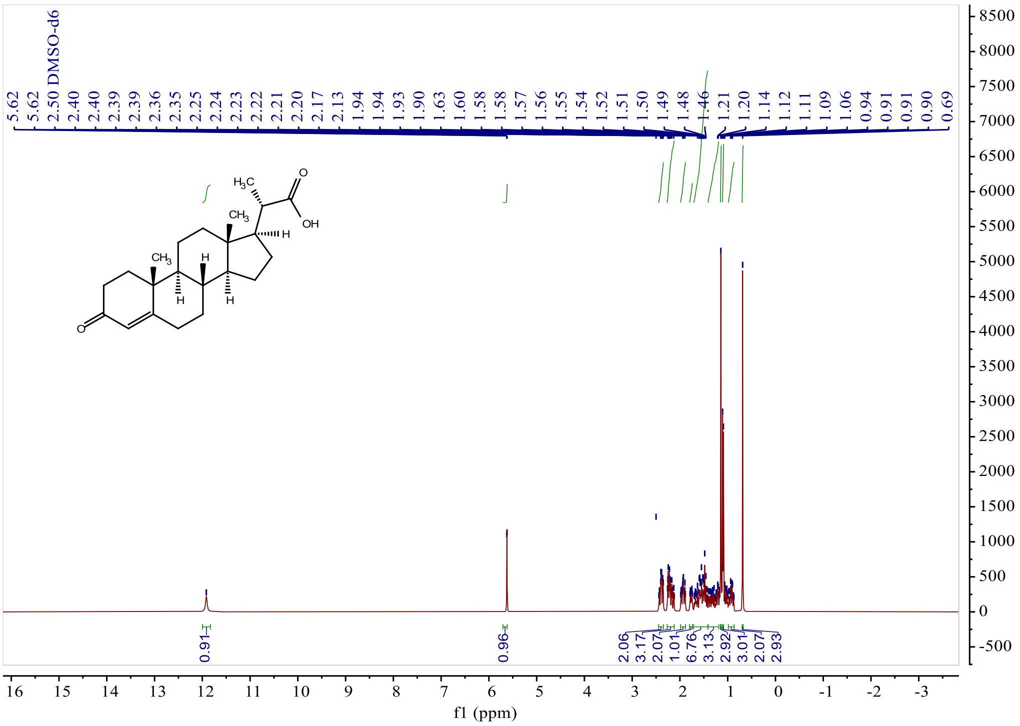

Figure S13 ^{1}H NMR (400 MHz, DMSO-d6) spectrum of **impurity 1-A**

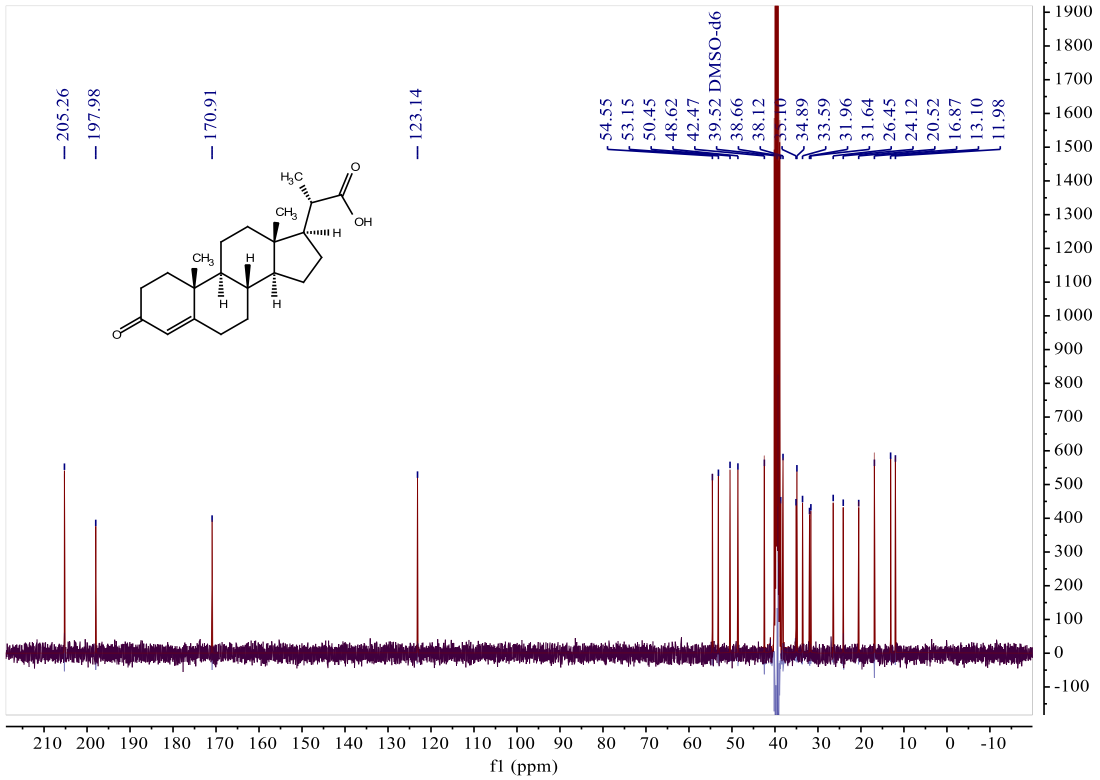

Figure S14 ^{13}C NMR (101 MHz, DMSO-d6) spectrum of **impurity 1-A**

7. ^{1}H NMR and ^{13}C NMR spectra of impurity 2-S

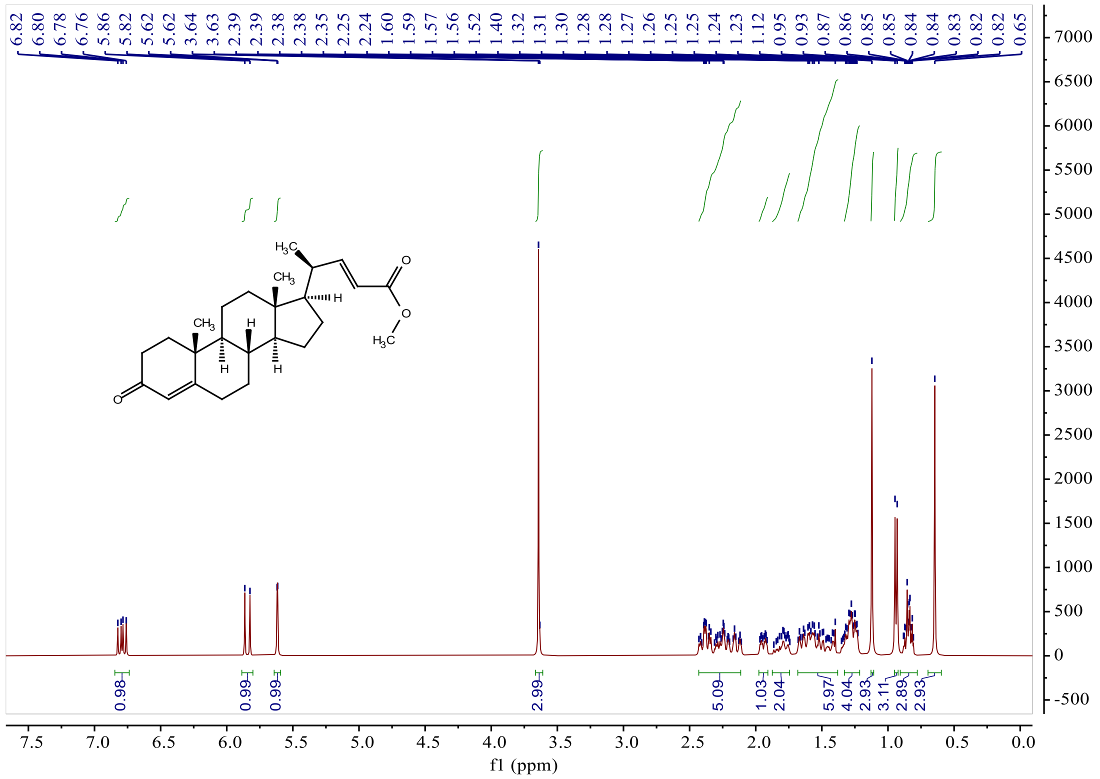

Figure S15 ^{1}H NMR (400 MHz, DMSO-d6) spectrum of **impurity 2-S**

8. ^{1}H NMR and ^{13}C NMR spectra of impurity 2-Z

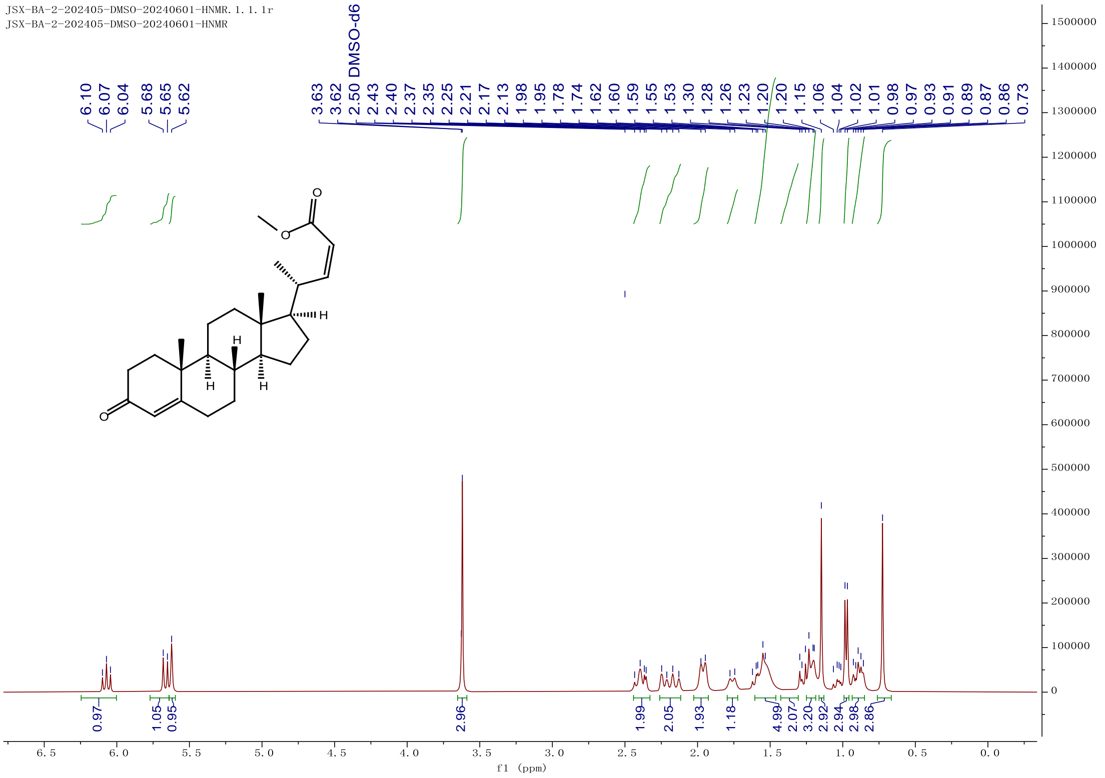

Figure S16 ^{1}H NMR (400 MHz, DMSO-d6) spectrum of **impurity 2-S**

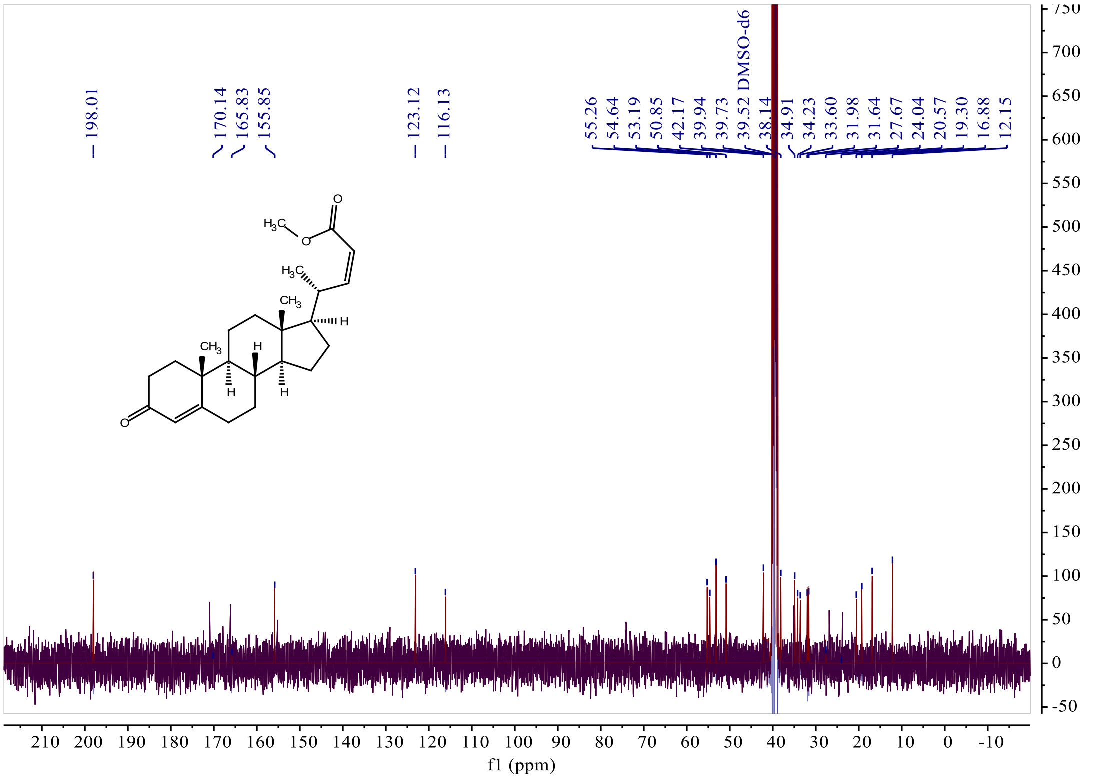

Figure S17 ^{13}C NMR (101 MHz, DMSO-d6) spectrum of **impurity 2-Z**

9. Mass analysis and LC-MS results for all the compounds and impurities

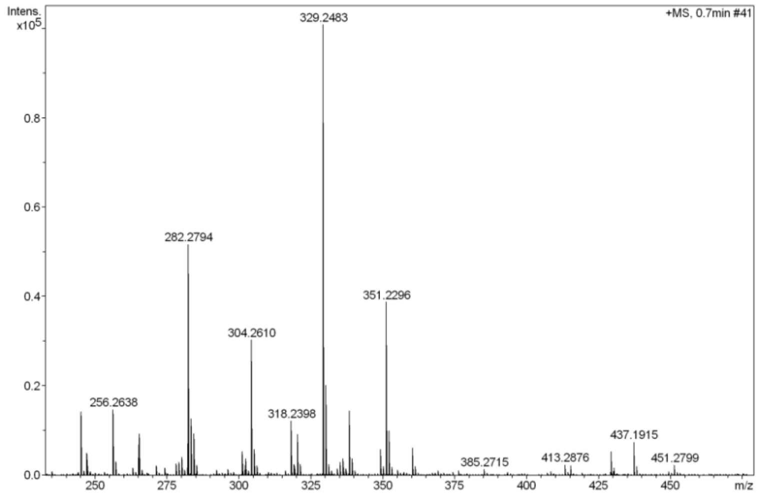

Figure S18 MS plot of **compound 1**

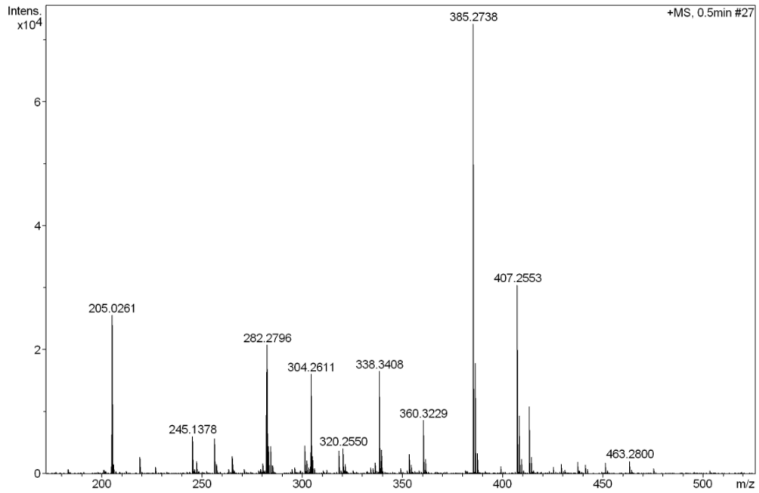

Figure S18 MS plot of **compound 2**

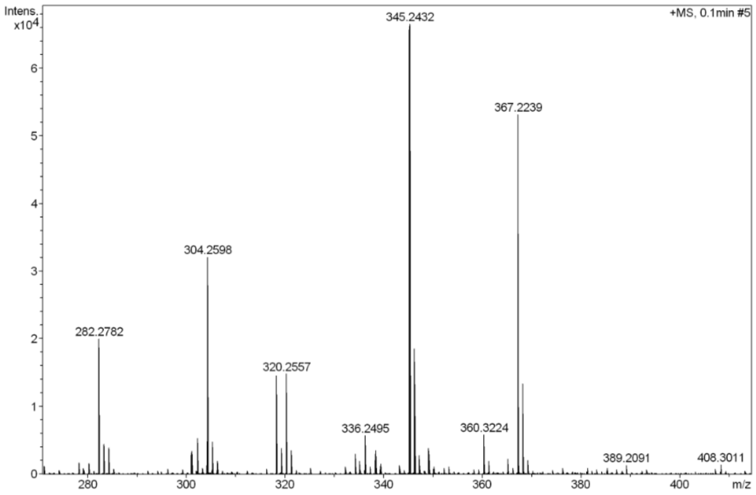

Figure S19 MS plot of **impurity 1-A**

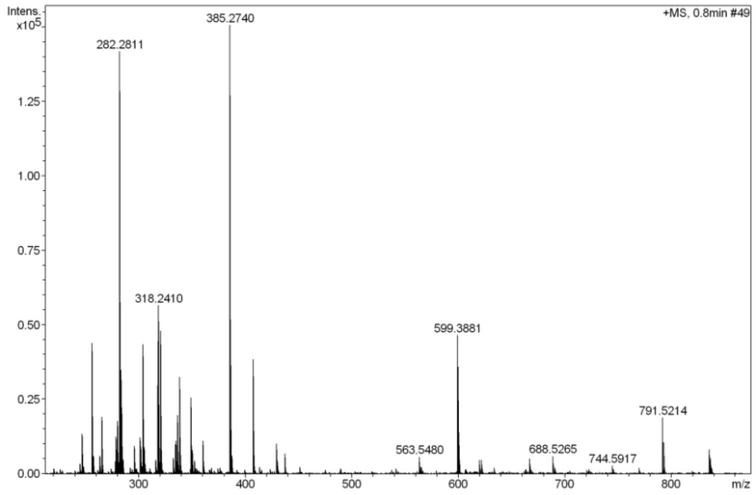

Figure S20 MS plot of **impurity 2-S**

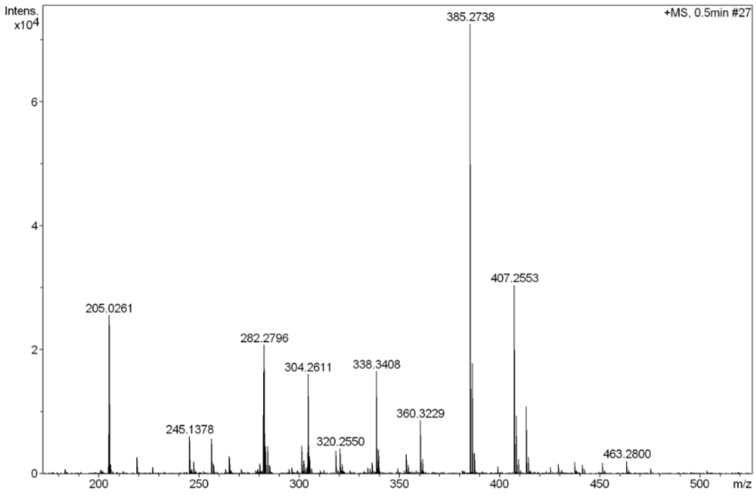

Figure S21 MS plot of **impurity 2-Z**

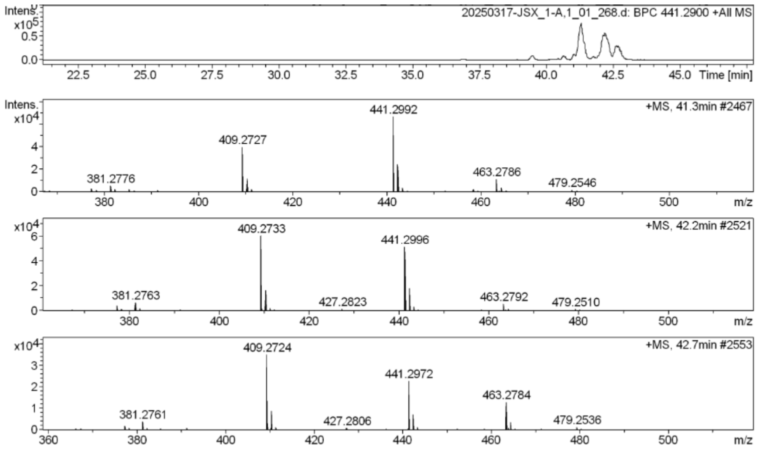

Figure S22 LC-MS plot of **impurity 2-D**
